# *The Adult Hip—Hip Preservation Surgery*. Edited by John Clohisy, Paul Beaulé, Craig Della Valle, John Callaghan, Aaron Rosenberg and Harry Rubash

**DOI:** 10.1093/jhps/hnu007

**Published:** 2014-08-29

**Authors:** Allston J. Stubbs

**Affiliations:** Wake Forest School of Medicine, Medical Center Boulevard, Winston-Salem, North Carolina, USA

At the dawn of the 21st century, hip preservation has been the *topic du jour* in orthopaedic surgery. *The Adult Hip—Hip Preservation Surgery* is an excellent compilation of the leading concepts regarding the evolution and revolution of this growing field. Drs. Clohisy, Beaulé, Dr. Della Valle *et al* have assembled a distinguished group of hip thought leaders and organized their chapters in a monograph that is clear, concise, and cogent.

The book is presented in nine sections across 61 distinctive chapters covering the basics such as the *Patient History and Exam* by Dr. Hal Martin to the more esoteric such as *Vascularized Grafting for Femoral Head Osteonecrosis* by Dr. Lodha and Dr. Wysocki. The use of descriptive text is well balanced with colourful artwork, sharp radiographs, and organized tables. The authors and editors have wisely avoided gross redundancy in both words and figures among the diverse chapter layout.

A distinguishing chapter is Chapter 1, *History of Hip Joint Preservation Surgery*, by Dr. Joseph Schatzker. This chapter adroitly and thoroughly takes us through the historical challenge faced by the surgeon presented with hip disease, and the theoretical and empirical realizations of the 20th century orthopaedic surgeon. Dr. Schatzker is wise to avoid the technical controversies and dilemmas addressed later in the book and carefully lays out the mechanical paradigms familiar to all who have worked in the field of hip preservation.

The book remains true to the foundation of hip disease by carefully building a case for anatomic and clinical understanding. Modern concepts such as genetics and cartilage imaging are included as well as the essentials of plain film radiographic evaluation. The additional areas of Computed Tomography (CT) and Magnetic Resonance Imaging (MRI) are included as separate chapters in the Patient Evaluation Section, but the concepts are not as thoroughly presented as one would expect. Readers looking for advanced theory on hip modeling and femoroacetabular impingement will find Chapter 39 by Dr. Ecker, Dr. Puls and Dr. Siebenrock to be exciting in its current scope and potential as the basis for future technology.

Although the separation of open and arthroscopic technical theory and application has been a goal of earlier hip preservation texts, the editors have done an admirable job of emulsifying a traditionally oil and water proposition. This success stems from the fact that considerable diligence has been made to recognize various perspectives on hip disease across a continuum of opportunity to best treat the patient. Chapter 44: *Combined Hip Arthroscopy and Mini-Anterior Hueter Approach for the Treatment of Femoroacetabular Impingement* by Drs. Lee and Beaulé is an example of this equilibrium. Additional efforts are made within the clinical case examples of authors’ methods to combine arthroscopy and open techniques to achieve desired clinical outcomes.

Although femoroacetabular impingement (FAI) surgery has been a dominant theme in the hip preservation world over the last 15 years, the editors have carefully avoided an FAI treatise in their work. Section 6 focuses on General Joint Preservation themes such as rehabilitation, treatment failures, and arthroplasty after hip preservation surgery. Section 7 is dedicated to osteonecrosis and the advanced nonoperative and operative treatment options. Finally, Sections 8 and 9 close out the book with chapters on the post-traumatic hip, synovial disorders and sports hip conditions.

In conclusion, *The Adult Hip—Hip Preservation Surgery* is a professionally done book in a quickly expanding area of orthopaedic surgery. Experienced hip surgeons as well as those new to the field will find it a valuable resource for both theory and technical advice. The editors and authors should be commended on their efforts to unite hip preservationists across the arthroscopic and open surgical spectrum. Readers will be pleased to find this monograph available as a print copy for their bookshelf or electronic copy for their portable e-readers.

